# The combined rapid detection and species-level identification of yeasts in simulated blood culture using a colorimetric sensor array

**DOI:** 10.1371/journal.pone.0173130

**Published:** 2017-03-15

**Authors:** Nabin K. Shrestha, Sung H. Lim, Deborah A. Wilson, Ana Victoria SalasVargas, Yair S. Churi, Paul A. Rhodes, Peter J. Mazzone, Gary W. Procop

**Affiliations:** 1 Department of Infectious Diseases, Medicine Institute, Cleveland Clinic, Cleveland, Ohio, United States of America; 2 Department of Clinical Pathology, Pathology and Laboratory Medicine Institute, Cleveland Clinic, Cleveland, Ohio, United States of America; 3 Specific Technologies, Mountain View, California, United States of America; 4 Department of Pulmonary and Critical Care Medicine, Respiratory Institute, Cleveland Clinic, Cleveland, Ohio, United States of America; Leibniz Institut - Deutsche Sammlung von Mikroorganismen und Zellkulturen GmbH, GERMANY

## Abstract

**Background:**

A colorimetric sensor array (CSA) has been demonstrated to rapidly detect and identify bacteria growing in blood cultures by obtaining a species-specific “fingerprint” of the volatile organic compounds (VOCs) produced during growth. This capability has been demonstrated in prokaryotes, but has not been reported for eukaryotic cells growing in culture. The purpose of this study was to explore if a disposable CSA could differentially identify 7 species of pathogenic yeasts growing in blood culture.

**Methods:**

Culture trials of whole blood inoculated with a panel of clinically important pathogenic yeasts at four different microorganism loads were performed. Cultures were done in both standard BacT/Alert and CSA-embedded bottles, after adding 10 mL of spiked blood to each bottle. Color changes in the CSA were captured as images by an optical scanner at defined time intervals. The captured images were analyzed to identify the yeast species. Time to detection by the CSA was compared to that in the BacT/Alert system.

**Results:**

One hundred sixty-two yeast culture trials were performed, including strains of several species of *Candida (Ca*. *albicans*, *Ca*. *glabrata*, *Ca*. *parapsilosis*, and *Ca*. *tropicalis*), *Clavispora (*synonym *Candida) lusitaniae*, *Pichia kudriavzevii* (synonym *Candida krusei)* and *Cryptococcus neoformans*, at loads of 8.2 × 10^5^, 8.3 × 10^3^, 8.5 × 10^1^, and 1.7 CFU/mL. In addition, 8 negative trials (no yeast) were conducted. All negative trials were correctly identified as negative, and all positive trials were detected. Colorimetric responses were species-specific and did not vary by inoculum load over the 500000-fold range of loads tested, allowing for accurate species-level identification. The mean sensitivity for species-level identification by CSA was 74% at detection, and increased with time, reaching almost 95% at 4 hours after detection. At an inoculum load of 1.7 CFU/mL, mean time to detection with the CSA was 6.8 hours (17%) less than with the BacT/Alert platform.

**Conclusion:**

The CSA combined rapid detection of pathogenic yeasts in blood culture with accurate species-level identification.

## Introduction

The presence of microorganisms in the bloodstream is a significant clinical finding, and always has an impact on clinical decision making. This fact makes blood culture an essential part of any clinical microbiology laboratory, and has ensured that as newer rapid diagnostic technologies are developed, their application to detect and identify microorganisms in blood cultures is examined. The current standard methodology for blood cultures involves incubation in automated blood culture systems that monitor for growth of microorganisms based on a colorimetric or fluorometric detection of CO_2_, followed by Gram staining and appropriate subculturing on solid media, followed by identification and susceptibility testing based on mass spectrometry and biochemical testing performed in other instruments.

Many innovations have attempted to identify microorganisms sooner once blood culture bottles provide a positive signal. These have included fluorescence *in situ* hybridization (FISH) [1] for specific microorganisms, broad-range polymerase chain reaction (PCR) with melt-curve analysis [2] or sequencing [3], matrix assisted laser desorption ionization-time of flight mass spectroscopy (MALDI-TOF MS) [4–6], and PCR coupled to electrospray ionization mass spectrometry (PCR/ESI-MS) [7]. Broad-range PCR for the identification of microbial pathogens directly from blood has been evaluated as an alternative rapid identification method, but this has had variable results [8–10]. Several of these technological advancements have been shown to reduce the time to bacterial identification once blood culture bottles turn positive. However, all these innovations still require additional processing after blood culture bottles signal positive.

It has recently been demonstrated that prokaryotic microorganisms (e.g. bacteria) produce species-specific profiles of the volatile organic compounds (VOCs) which are emitted as byproducts of their metabolism during growth in culture, and that these profiles can be characterized with standard analytical instruments, such as gas chromatography-mass spectroscopy (GC-MS) [11]. The elaboration of these compounds produces the recognizable odors associated with specific bacteria [12]. However, mass spectroscopy based techniques require expensive equipment, special training and are reliant, under current FDA-approved protocols, upon the testing of isolated colonies from culture or subculture, which is time consuming [13].

Recent advances in various array based sensor (electronic nose) technologies opened promising new opportunities in biomedical and industrial research with the capability of identifying individual compounds and complex mixtures of VOCs using pattern-recognition algorithms [13–15]. Among these, a disposable colorimetric sensor array (CSA) has been shown to identify a panel of pathogenic bacteria with great accuracy [16–19]. The promise of this low cost technology is that it is rapid, with identification obtained automatically during culture, saving time, labor, and cost. This sensor technology has recently been shown to accurately detect and identify pathogenic bacteria in blood culture bottles [20]. Yeasts, eukaryotes with a far different cellular architecture, are also important bloodstream pathogens and any blood culture system must be able to detect and identify yeasts accurately.

The purpose of this study was to conduct a proof-of-principle to determine if a disposable CSA could detect and identify different yeasts growing in blood cultures, establishing the method for eukaryotes, and to quantify the speed and accuracy with which this could be done.

## Materials and methods

### Test panel of yeasts and sample preparation

A selection of American Type Culture Collection (ATCC) strains of clinically important pathogenic yeasts was made for inclusion in a test panel. The panel consisted of *Candida* species (*Ca*. *albicans* ATCC 10231, *Ca*. *albicans* ATCC 14053, *Ca*. *glabrata* ATCC 15126, *Ca*. *parapsilosis* ATCC 22019, and *Ca*. *tropicalis* ATCC 13803), *Clavispora* (synonym *Candida) lusitaniae* ATCC 34449, *Pichia kudriavzevii* (synonym *Candida krusei)* ATCC 6258, and *Cryptococcus neoformans* species complex ATCC 14116.

The included yeast species were grown on potato dextrose agar. A few colonies were suspended in sterile normal saline and the turbidity adjusted to a 2.0 McFarland standard. Serial 1:100, 1:100, and 1:50 dilutions were performed to obtain 1:100, 1:10000, and 1:500000 dilutions of the 2.0 McFarland suspension, respectively. 0.5 mL of the 2.0 McFarland suspension and each of these diluted suspensions were inoculated into 9.5 mL of discarded packed red blood cells (henceforth referred to as “blood”) obtained from the Cleveland Clinic blood bank. 50 μL of the penultimate dilutions were also plated on blood agar plates for verification of colony counts.

The blood samples spiked with several clinically important species of yeasts at these four different loads, which span clinically relevant loads, were used for testing.

### Culture methods

Spiked blood samples were cultured in BacT/Alert^®^ FA plastic blood culture bottles (bioMérieux Inc., Durham, NC, USA). This was done by inoculating 10 mL of a spiked blood sample into a blood culture bottle containing 40 mL of growth medium.

### Colorimetric sensor array system

**Fig 1:**
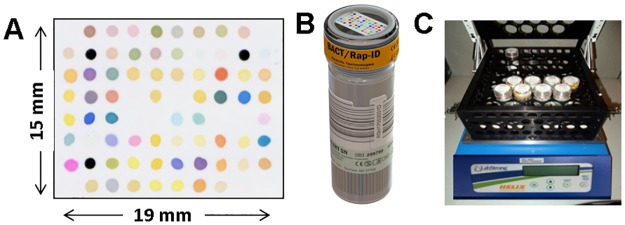
Colorimetric Sensor Array (CSA) system. (A) CSA. (B) CSA-embedded blood culture bottle. (C) CSA prototype blood culture instrument.

The CSA system has two major components: a disposable sensor and a sensor reader containing an optical scanner. The CSA is a disposable array of cross-responsive indicators that collectively detects and discriminates a wide range of chemical analytes and complex mixtures [21, 22]. General array manufacturing procedures have been previously reported [23]. The CSA used with the blood culture bottle contained 73 indicators to cover a diverse portfolio of dye-analyte interactions, and 3 black fiducials (Fig 1A).

The sensor was mounted inside a transparent cap designed to fit a blood culture bottle while allowing a sufficient air gap between the sensor and the cap to ensure adequate diffusion of headspace gases over the sensor. The cap of a standard aerobic blood culture bottle (bioMérieux) was replaced with this specially designed sensor cap (Fig 1B). Placing the sensor inside the bottle cap allowed headspace gases to be monitored continuously during incubation of cultures without disrupting the gas mixture.

A CSA prototype blood culture instrument was specifically designed to hold blood cultures at a set temperature, provide gentle broth agitation to stimulate growth, and allow sensor data to be collected in real time. This instrument held up to 36 bottles in a vertical orientation to prevent broth from splashing the sensor during shaking. Sensor data collection was done by a commercial flatbed scanner (EPSON Perfection V600) placed face-down over the bottles to image the sensors through the transparent bottle caps at 20 minute intervals for the duration of incubation. From each image, red, green and blue (RGB) color values were acquired for each indicator in the sensor array. The entire assembly was placed inside an incubator set at 37°C (Fig 1C). Between images, the bottle assembly was agitated at 200 rpm by a computer-controlled orbital shaker.

### Incubation of blood cultures

Blood samples spiked with yeast species were inoculated in CSA-embedded blood culture bottles and incubated in the CSA prototype blood culture instrument (Fig 1C) for 72 hours. Additionally, samples were also inoculated in standard blood culture bottles (i.e. intact BacT/Alert blood culture bottles) and incubated in a BacT/Alert blood culture monitoring system in the standard manner for blood culture incubation in the clinical laboratory. This was done to compare time to detection by the CSA method and the standard blood culture method. The standard blood culture method consisted of incubation for 96 hours or until a positive signal was detected, whichever occurred sooner. A sample of spiked blood was also cultured on TSA II 5% sheep blood agar as a culture purity check that the sample inoculated was not contaminated. Each yeast species was tested multiple times as summarized in the results section. Each test was considered one trial.

### CSA image processing

The CSA was imaged by a scanner every 20 minutes after inoculation. For each image, for each of the 73 indicators, the median of all pixels within a 10-pixel-radius disk centered in each of the indicator spots was calculated, providing RGB values for each indicator, thereby yielding a 219-dimensional vector of RGB colors (73 indicators x 3 colors). An image captured at 4 hours after initial exposure was defined as the reference image, and its corresponding color vector the reference vector. All images captured prior to the reference image were ignored. The choice of the 4-hour image as the reference image gave most indicators enough time to equilibrate in response to the gas released from the growth medium and occupying the headspace region. Following generation of the reference image, a 219-dimensional color vector was generated for each sample every 20 minutes. From each of these vectors, the reference vector was subtracted, yielding color difference vectors for each 20-minute time point for each sample. As no fungal emissions were relevant at the 4 hour mark, each color difference vector described depicted the *change* in the sensor indicator colors after the 4 hour period allocated for the equilibration of the sensor array with the emissions of blood plus growth medium.

### Data analysis

#### Detection of fungal growth by the CSA

For each trial, the series of vectors yielded a time series of color change patterns for the indicators in the sensor array. Color changes in various indicators facilitated detection of fungal growth. A plot of color change against time provided separate kinetic profiles for red, green and blue values for each indicator. Color changes in different indicators occurred at different rates. A detection was made by any indicator when the slope for that detector crossed its detection threshold. Using the kinetic profiles of all indicators, a determination of fungal growth was made by defining fungal growth detection as a response 2 standard deviations from response to control (blood plus growth medium without microorganisms) of the fastest responding indicators.

#### Species identification by species-specific colorimetric patterns

Each 219-dimensional color vector was standardized to have element-by-element zero mean and unit standard deviation across all trials. Species classification was done by training a support vector machine (SVM) on the time series of the rate of color changes, and pairwise comparisons were used to separate multiple classes [24]. Using 10 repetitions of 10-fold stratified cross validation, the sensitivity of species identification was determined at the time of detection and every subsequent hour for 8 hours.

#### Comparison of time to detection

For all available paired trials (i.e. those trials for which results from both the CSA and the standard incubation method were available), time to detection by the CSA method was compared with the time to detection by the standard method. Detection by the CSA method was described above. For the BacT/Alert system, detection was defined as the time a bottle yielded a positive signal. For both standard CO_**2**_-based detection (the BacT/Alert system) and the CSA method, the time to detection was defined as the time from start of blood culture incubation to the time of detection of a positive signal.

## Results

A total of 180 trials including 8 controls were conducted with the panel of yeasts. Excluding trials rejected due to bacterial contamination (6), lack of growth on purity plates (1), and machine inoculation or assembly errors (3), the microorganism strain distribution for the remaining 170 included trials was as follows: *Ca*. *albicans* ATCC10231 (13), *Ca*. *albicans* ATCC 14053 (12), *Ca*. *glabrata* ATCC 15126 (35), *Ca*. *parapsilosis* ATCC 22019 (13), *Ca*. *tropicalis* ATCC 13803 (16), *Cl*. *lusitaniae* ATCC34449 (15), *P*. *kudriavzevii* ATCC 6258 (19), *Cr*. *neoformans* species complex ATCC 14116 (39), and controls (8). The 162 included trials with yeasts had cultures done with 10 mL of blood inoculated with the 2.0 McFarland suspensions (40 trials), 1:100 dilutions (41 trials), 1:10000 dilutions (40 trials), and 1:500000 dilutions (41 trials).

Based on colony counts of the 1:10000 dilutions, the calculated loads (mean ± SD) of the original 2.0 McFarland suspensions for the yeast species were 9.86 ± 4.64 × 10^6^ CFU/mL (*Ca*. *albicans*), 23.62 ± 9.47 × 10^6^ CFU/mL (*Ca*. *glabrata*), 9.20 ± 2.55× 10^6^ CFU/mL (*Ca*. *parapsilosis*), 27.10 ± 22.41× 10^6^ CFU/mL (*Ca*. *tropicalis*), 16.33 ± 1.44 × 10^6^ CFU/mL (*Cl*. *lusitaniae*), 4.45 ± 1.11 × 10^6^ CFU/mL (*P*. *kudriavzevii*), and 18.98 ± 1.81× 10^6^ CFU/mL (*Cr*. *neoformans*). The highest dilution (1:500000) would thus have had mean yeast loads ranging from 8.9 to 54.2 CFU/mL for the different yeast species. With the additional 1:20 dilution occurring when inoculated into blood, the blood samples spiked with the least concentrated yeast suspensions would have had yeast loads (mean ± SD) as follows: 1.04 ± 0.55 CFU/mL (*Ca*. *albicans*), 2.36 ± 0.95 CFU/mL (*Ca*. *glabrata*), 0.92 ± 0.26 CFU/mL (*Ca*. *parapsilosis*), 2.71 ± 2.24 CFU/mL (*Ca*. *tropicalis*), 1.63 ± 0.12 CFU/mL (*Cl*. *lusitaniae*), 0.44 ± 0.10 CFU/mL (*P*. *kudriavzevii*), and 1.78 ± 1.07 CFU/mL (*Cr*. *neoformans*). The mean inoculum loads (yeast loads in the spiked blood samples) at the four dilutions would have been 8.2 × 10^5^, 8.3 × 10^3^, 8.5 × 10^1^, and 1.7 CFU/mL.

### Color difference patterns for different yeasts

**Fig 2:**
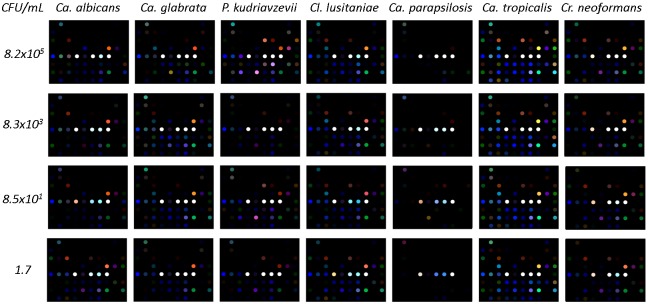
Representative color difference maps for the yeasts tested.

Fig 2 shows representative color difference maps for the different yeasts, at the four inoculum loads. For display purposes only, the color range shown in the difference maps was expanded from RGB values of 4–35 to 0–255. The color difference maps at the end of trial for seven different yeast species at different inoculum loads were generated by subtracting the mean RGB of each color spot from a baseline image taken at 4 hours. In this way, both the initial color of each indicator and its response in equilibrating to the volatiles emitted by growth medium and blood were neglected and the sensor response was the change produced by the volatiles emitted by growing microorganisms. The CSA fingerprint was species specific, and the pattern was highly consistent across inoculum load across the 500000-fold range of loads tested.

### Kinetic profiles of different yeast species

**Fig 3:**
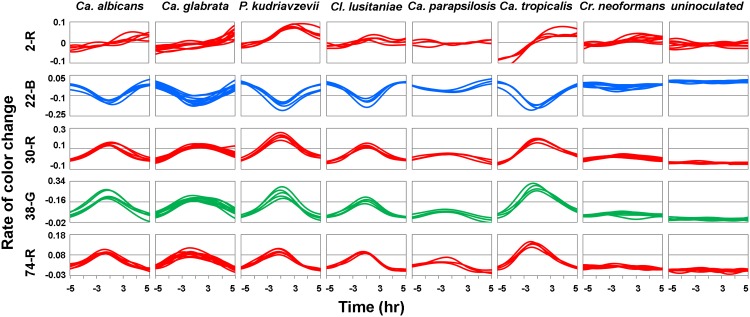
Sensor response time derivatives for selected indicator channels for multiple trials for each yeast species.

Fig 3 shows sensor response time derivatives for selected indicator “channels” (red, green and blue color values are each distinct channels, so 3 channels for each indicator and 219 for all 73 indicators in the sensor array) in the array, for multiple trials for each species. Each row represents the time derivative of the color response of a single “channel” (for example, the top row of traces, labelled 2-R, depicts the rate of change in the red component of indicator 2). The five selected indicator responses show kinetic profiles of 7 species of yeast inoculated at a mean yeast load of 1.7 CFU/mL. RGB-color-change slopes between ±5 hours of the detection time are shown, with each line representing an independent trial. A straight line indicates that the indicator is not changing color (no growth of yeast). A positive slope indicates that an indicator color is getting lighter, a negative slope indicates that the color is getting darker (black color is represented as 0,0,0 in an RGB space, while white color is represented as 255,255,255). The magnitude of the slope shows how fast the color is changing. *Cr*. *neoformans* shows little response for these indicators in comparison to other species, so it is plotted on a smaller scale. The figure shows that repeated trials for the same species generated similar kinetic profiles for a given sensor (i.e. the individual replicate traces were overlapping), an indication that the individual sensor responses were repeatable. These experiments suggest that kinetic profiles for different yeasts are species specific, suggesting the potential for differentiating between yeasts based on the data acquired through the sensor array.

### Clustering of sensor responses

**Fig 4:**
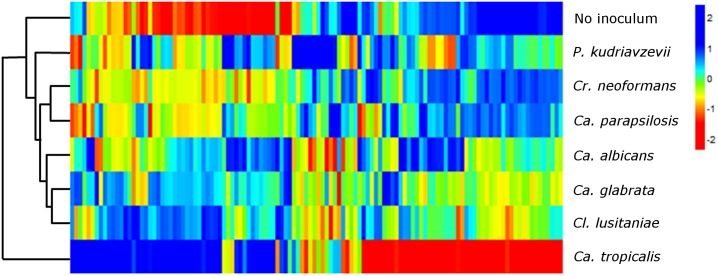
Heat map of mean CSA sensor responses for 7 species of yeast and uninoculated control.

To utilize all 219 sensor channels to more fully visualize the species-specific sensor patterns produced during growth in culture, we sampled the rate of color change for each indicator 3 hours after fungal detection. Fig 4 shows a heat map used to visualize the rates of color change. For each trial, a 219-feature vector of the 73 indicators was created using RGB-color-change slopes at 3 hours after the detection time. Each feature was standardized to have zero mean and unit variance across all trials, and assigned a column in the heat map. Feature columns were ordered by Euclidean-distance clustering, and heat map rows corresponded to centroid kinetic profiles for each species. The heat map revealed unique yeast VOC signatures for the 7 species of yeast in blood cultures and sterile blood cultures. Note that each row is a distinct pattern, indicating that the different yeast species had distinct VOC signatures.

### Sensitivities of yeast detection and species identification

The CSA detected the presence of yeast in blood culture bottles inoculated with spiked blood with 100% sensitivity and 100% specificity. Thus all 162 trials with spiked blood culture samples were identified as positive and all 8 trials without spiked blood were identified as negative. The majority of species could be identified immediately after detection, and the sensitivity of identification improved over the hours following detection. Table 1 shows the sensitivity of species identification over time starting from the time of detection of a positive signal. While *Cr*. *neoformans* could be identified immediately after detection with 93.1% sensitivity, most other yeast species had moderate sensitivity for identification at that time, with an overall sensitivity of 72.1% at detection. However, the sensor pattern rapidly ripened after initial detection, and within 2 hours after detection, the mean sensitivity for identification for all species was 90.4% (95% confidence interval 84.8–94.3%). By 4 hours after CSA detection of growth the mean sensitivity for species identification of the different yeast species reached 94.4%, ultimately reaching 96.6% (95% CI; 92.5–98.7%) a few hours thereafter.

**Table 1:** Sensitivity for species identification with CSA as a function of time following microorganism detection.

Species	n	0 hr	+1 hr	+2 hr	+3 hr	+4 hr	+5 hr	+6 hr	+7 hr	+8 hr
Uninoculated	8	100.0	100.0	100.0	100.0	100.0	100.0	100.0	100.0	100.0
*Ca*. *albicans*	25	67.2	78.0	84.8	84.8	89.6	86.4	89.6	92.0	94.4
*Ca*. *glabrata*	35	63.4	86.9	87.4	91.7	92.0	94.6	96.0	96.9	96.9
*Ca*. *parapsilosis*	14	62.9	72.9	83.6	86.4	88.6	90.0	87.9	90.7	89.3
*Ca*. *tropicalis*	15	68.0	93.3	96.0	96.7	99.3	94.7	96.0	98.0	100.0
*Cl*. *lusitaniae*	15	55.3	76.7	87.3	86.7	91.3	90.7	93.3	97.3	99.3
*P*. *kudriavzevii*	19	67.4	76.8	85.8	94.2	94.7	94.7	94.7	95.3	95.3
*Cr*. *neoformans*	39	93.1	97.4	98.0	99.7	100.0	99.5	98.2	97.2	97.4
**Total**	**170**	**72.2**	**85.3**	**90.4**	**92.5**	**94.4**	**93.8**	**94.5**	**95.9**	**96.6**

Sensitivity was calculated with a support vector machine using 10 repeats of 10-fold cross validation. Note that initial detection (here Time 0 hr) occurred a mean of 6.3 hours ahead of that in the BacT/Alert system, so the data reflect that *Candida* species identification reached 94.4% before detection occurred on that widely used clinical platform.

### Time to detection with CSA compared to BacT/Alert system

**Fig 5:**
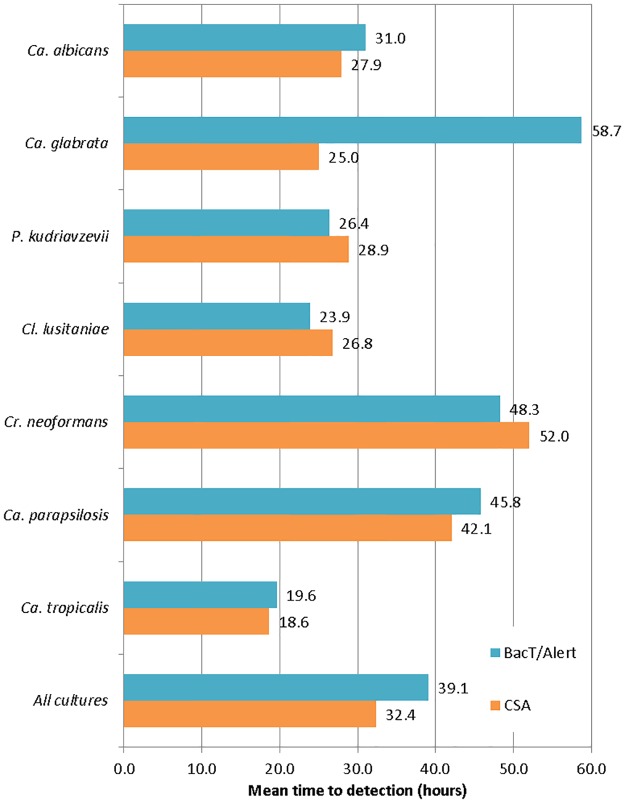
Comparison of time to detection of yeast species by the CSA and standard (BacT/Alert) methods.

At lower loads, the CSA method detected the presence of yeast faster than the BacT/Alert method did. For all species, at an inoculation load of 1.7 CFU/mL, the CSA method detected the presence of yeast a mean of 6.8 hours (17%) earlier than the BacT/Alert method, incubated simultaneously (Fig 5).

## Discussion

This proof-of-concept study suggests that the CSA described may be able to combine the more rapid detection of pathogenic yeasts growing in blood culture bottles with the identification of microorganisms at the species level. This is the first report of a CSA being utilized to identify the species of a eukaryote, and supports the conjecture that all microorganisms growing in culture emit a unique pattern of volatiles as byproducts of their metabolic activity and that volatile-sensing CSA’s can be used to “fingerprint” the identify of all microorganisms across kingdoms. The time to detection of yeast in blood culture by the CSA compared favorably to that of detection in the BacT/Alert blood culture monitoring system. It is to be noted that these were findings with growth media optimized for the BacT/Alert system. With optimization of the growth medium for the CSA, the advantage of the sensor array may be even greater. A study of 152 episodes of candidemia, which used a lysis-centrifugation blood culture method, found that the fungal burden in the first positive blood culture ranged from 0.1 CFU/mL to > 1000 CFU/mL, with 68% having yeast loads ≤ 5 CFU/mL [25]. The range of loads examined in our study encompassed this clinically relevant range. At the lowest load tested (1.7 CFU/mL), the CSA system detected growth of fungi at a mean of 6.8 hours (17%) faster than the BacT/Alert system. Within several hours after detection the sensitivity for species identification by the CSA reached approximately 95%; thus highly accurate species identification was possible before a positive signal was even identified in the BacT/Alert system.

The study is limited by its small sample size. Larger studies with validation in external cohorts, and ultimately performance with blind clinical samples, will be necessary to demonstrate that such differentiation can be done reliably in clinical settings. Accurate evaluations of the sensitivity and specificity of such testing will require more species of pathogenic yeasts and evaluation of many specimens of each species, including clinical isolates. With respect to the occurrence of mixed specimens, either from a genuinely polymicrobial infection or contamination of sample during collection or otherwise, we expect but have not demonstrated that samples would produce a more rapid positive than legacy methods, but the presumably complex and unrecognizable CSA signatures of mixed (or novel species) samples would yield an “unclassifiable” result and recommendation to proceed with subculture.

In routine testing in clinical microbiology laboratories, a positive signal from a standard blood culture monitoring system does not identify the pathogen but only signals that the blood culture is positive. Additional steps are necessary to identify the microorganism growing. The advantage of CSA for blood cultures is that it can identify the growing microorganism at the species level without any additional testing. That had been shown for bacteremia, and now we have extended this novel paradigm to eukaryotes. This study suggests that the CSA can detect and identify yeast species in blood cultures faster than detection alone is rendered in the BacT/Alert blood culture system. Moreover, because species identification occurs during culture and can be performed by automated camera imaging of the sensor combined with automated match to a library of pathogenic yeast “fingerprints”, identification therefore can occur in an automated fashion around the clock. If a lab is unstaffed in the middle of the night, the presence of yeast infection along with a 95% accurate species identification can be transmitted to the lab information system, or otherwise relayed to the clinical unit.

Rapid identification of yeasts to species level can help with appropriate antimicrobial selection. For example, many institutions use an echinocandin for empiric treatment of fungal infections, but this would be an inappropriate selection for a patient with cryptococcosis given the innate resistance of *Cr*. *neoformans* to these agents. Similarly, identification of *Ca*. *glabrata* or *P*. *kudriavzevii* would indicate that fluconazole (a commonly used antifungal for *Candida* infections) would not be the appropriate treatment.

In conclusion, this proof-of-principle study shows that clinically important yeasts growing in blood culture have differences in their volatile organic compound signatures that allow their identification by a disposable CSA faster than current methods perform detection alone. If borne out by larger studies and when challenged with clinical samples, the low cost, low labor and automated nature of this testing platform could have widespread application for identifying fungi causing bloodstream infections.
